# Fetal microchimerism in kidney biopsies of lupus nephritis patients may be associated with a beneficial effect

**DOI:** 10.1186/s13075-015-0615-4

**Published:** 2015-04-15

**Authors:** Greiciane MS Florim, Heloisa C Caldas, Julio CR de Melo, Maria Alice SF Baptista, Ida MM Fernandes, Marcela Savoldi-Barbosa, Gustavo H Goldman, Mario Abbud-Filho

**Affiliations:** Department of Medicine, Laboratory of Immunology and Experimental Transplantation (LITEX), Avenida Brigadeiro Faria Lima 5416, 15090-000 Sao Jose do Rio Preto, Brazil; Division of Nephrology, Department of Medicine, Hospital de Base, Medical School of Sao Jose do Rio Preto (FAMERP), Avenida Brigadeiro Faria Lima 5416, 15090-000 Sao Jose Rio Preto, Brazil; Departament of Pharmacology, Faculty of Sciences Ribeirão Preto Pharmaceutical, University of Sao Paulo (FCFRP/USP), Avenida do Café s/n, 14040-903 Ribeirão Preto, Brazil; Institute of Urology and Nephrology, Rua Voluntários de São Paulo, 3826, 15015-200 Sao Jose Rio Preto, Brazil

## Abstract

**Introduction:**

Microchimeric male fetal cells (MFCs) have been associated with systemic lupus erythematosus, and published studies have further correlated MFC with lupus nephritis (LN). In the present study, we evaluated the frequency of MFC in the renal tissue of patients with LN.

**Methods:**

Twenty-seven renal biopsies were evaluated: Fourteen were from women with clinical and laboratory findings of LN, and thirteen were from controls. Genomic DNA was extracted from kidney biopsies, and the male fetal DNA was quantified using real-time quantitative polymerase chain reactions for the detection of specific Y chromosome sequences.

**Results:**

MFCs were detected in 9 (64%) of 14 of patients with LN, whereas no MFCs were found in the control group *(P* = 0.0006). No differences in pregnancy history were found between patients with LN and the control group. Significantly higher amounts of MFCs were found in patients with LN with serum creatinine ≤1.5 mg/dl. Furthermore, women with MFCs had significantly better renal function at the time of biopsy (*P* = 0.03). In contrast, patients with LN without MFCs presented with more severe forms of glomerulonephritis (World Health Organization class IV = 60% and class V = 40%).

**Conclusions:**

Our data indicate a high prevalence of MFCs in renal biopsy specimens from women with LN, suggesting a role for MFCs in the etiology of LN. The present report also provides some evidence that MFCs could have a beneficial effect in this disease.

**Electronic supplementary material:**

The online version of this article (doi:10.1186/s13075-015-0615-4) contains supplementary material, which is available to authorized users.

## Introduction

Renal involvement occurs in 50% to 70% of patients with systemic lupus erythematosus (SLE) and is a major cause of morbidity and mortality observed in this disease. Lupus nephritis (LN) has a direct impact on disease outcomes by causing damage to target organs, with 10% to 30% of SLE patients developing end-stage renal disease [1,2].

Several mechanisms have been reported to cause the loss of self-tolerance present in SLE and contribute to tissue injury and organ dysfunction, including heritable, epigenetic, environmental, hormonal and immune regulatory factors [3].

As SLE affects women at a tenfold greater incidence than men, and because these women present their first symptoms most often during their fertile years, authors of recent reports have postulated a role for microchimerism among the etiologic factors of SLE [4]. Pregnancy is the most important source of microchimeric cells, and the circulation of these cells between mother and fetus also causes maternal microchimerism. Therefore, it seems reasonable to speculate that microchimeric cells, especially those generated from male fetuses, could play a role in the development of SLE and LN [5].

There are several reports in which microchimeric male fetal cells (MFCs) have been described as being associated with immune-mediated diseases [6-9]. Although some authors have reported chimerism occurring twice as often in kidneys of patients with LN, few authors have correlated MFCs with LN, and these studies have often yielded inconsistent results [10-13].

We have previously reported that patients with SLE have more MFCs in the peripheral blood than healthy women [14]. In the present study, we evaluated the frequency of MFCs in the renal tissue of patients with LN and whether MFCs could be associated with pregnancy history and the type of kidney injury observed in biopsies.

## Methods

### Patients

Renal biopsies of 27 women were evaluated in this study: 14 from women with a diagnosis of LN (LN group) and 13 from women whose biopsies were performed for diagnosis of other types of glomerulopathies (control group) without a previous clinical history of autoimmune disease.

The inclusion criteria for both groups were having given birth to at least one male child, having received no blood transfusions and having had no previous organ transplants or abortions. No patient in the LN group had been undergone dialysis at the time of biopsy.

By using a personal interview and questionnaire, a detailed pregnancy history was taken from patients and control subjects to collect demographic data, especially the age at birth of first male child, the number of male pregnancies and the time since the birth of the first male child. To ensure that inclusion criteria were strictly followed, women without these data were not included in the study. The study was approved by the institutional ethics committee of the Medical School of Sao Jose do Rio Preto (FAMERP) (number 5863/2011) in accordance with current standards for human research, and informed consent of all patients was obtained.

### Diagnostic criteria

Patients with SLE were diagnosed according to the 1982 revised criteria of the American College of Rheumatology for the diagnosis of SLE [15]. Histological findings of LN from biopsies were classified according to the World Health Organization (WHO) classification scheme, as updated in 2003 by the International Society of Nephrology/Renal Pathology Society [16]. Clinical diagnoses of LN were made based on the finding of an “active” urinary sediment (proteinuria >1+, erythrocytes >5,000/ml) and/or serum creatinine ≥1.5 mg/dl and/or measured creatinine clearance ≤60 ml/min/73 m^2^ and/or 24-hour proteinuria >300 mg observed in at least two consecutive outpatient appointments.

Activity and chronicity indices were estimated based on the scoring system of Pollak *et al*. [17], as modified by Austin *et al*. [18].

### DNA extraction and real-time quantitative polymerase chain reaction for detection of Y chromosome sequences

Genomic DNA was extracted from kidney biopsies embedded in paraffin blocks using the RecoverAll™ Total Nucleic Acid Isolation Kit for formalin fixation and paraffin embedding (Ambion; Life Technologies, Carlsbad, CA, USA), according to the manufacturer’s instructions. Subsequently, the measurement of DNA concentration by spectrophotometry was conducted (NanoDrop 2000 spectrophotometer; NanoDrop Products, Wilmington, DE, USA) and the concentration of the genomic DNA samples was adjusted to 50 ng/μl.

The amount of male fetal DNA present was determined by real-time quantitative PCR (qPCR) through detection of specific Y chromosome sequences using the StepOnePlus™ Real-Time PCR System, version 2.2 (Applied Biosystems; Life Technologies).

The sex-determining region Y (SRY) TaqMan system consisted of a forward primer: 5′-CGC ATT CAT CGT GTG GTC TC-3′; a reverse primer: 5′-CTC TGA GTT TCG CAT TCT GGG-3′; and a probe: (VIC; Life Technologies) 5′-CGA TCA GAG GCG CAA GAT GGC TCT AG-3′ (tetramethylrhodamine) [19].

The SRY results were normalized using the threshold cycle values obtained for β-actin (endogenous control) amplified on the same plate [20].

The concentrations of male fetal DNA were expressed as genome equivalents. One genome equivalent was defined as the quantity of a particular DNA sequence present in one diploid male cell [19]. All samples were analyzed in duplicate.

### Statistical analysis

Categorical variables were compared with the use of the χ^2^ test. Continuous variables were compared with Student’s *t*-test and the nonparametric were compared with the Mann–Whitney *U* test. The Spearman correlation test was used to identify trends between different variables and the amount of MFCs. Multiple linear regression analysis was used to evaluate possible dependence of the amount of MFCs with continuous variables. The Spearman correlation test was used to identify trends between different variables and the amount of MFCs. All data analyses were performed using GraphPad Prism software (GraphPad Software, La Jolla, CA, USA). *P* < 0.05 was considered significant.

## Results

### Pregnancy history of patients with lupus nephritis and the control group

The LN and control groups showed no differences with regard to age at time of biopsy, age at birth of first male child, number of male pregnancies and time since birth of the first male child.

MFCs were detected in the renal biopsy specimens of 9 (64%) of 14 patients with a diagnosis of LN, whereas no MFCs were found in the renal biopsy samples of the control group (*P* = 0.0006, odds ratio = 46.6).

When we evaluated the amount of MFCs in the LN group, we observed a trend toward a lower proportion of MFCs in patients with a longer period of time since the birth of the first male child (>15 years), when the SLE diagnosis was made >5 years previously and among multiparous women, although none of these factors reached statistical significance. Interestingly, the proportion of MFCs was almost 100-fold higher among women presenting with better renal function at the time of biopsy (serum creatinine levels ≤1.5 mg/dl = 9.5 ± 11.2 *versus* >1.5 mg/dl = 0.12 ± 0.2; *P* = 0.026) (Table 1).

**Table 1:** **Amount of male fetal cells in renal biopsies from patients with lupus nephritis, pregnancy history and serum creatinine levels** ^**a**^

**Characteristic**	**LN group (n = 14)**	***P-value***
Time since birth of first male child (yr)		
1 to 15 (n = 5)	9.25 ± 12.37	NS
>15 (n = 9)	5.5 ± 9.6	
Time since SLE diagnosis (yr)		
1 to 5 (n = 5)	10 ± 13.6	NS
>5 (n = 9)	5.08 ± 8	
Number of male pregnancies		
1 (n = 0)	7.99 ± 10.75	NS
>1 (n = 4)	3.7 ± 7.5	
Serum creatinine (mg/dl)		
≤1.5 mg/dl (n = 10)	9.5 ± 11.2	0.026
>1.5 mg/dl (n = 3)	0.12 ± 0.2	

^a^LN, Lupus nephritis; NS, Not significant; SLE, Systemic lupus erythematosus. Data are given as mean ± standard deviation.

### Clinical and laboratory features of women with lupus nephritis, with and without male fetal cells in renal tissue

As 5 (36%) of 14 patients with LN did not present with MFCs in their renal tissue, we sought to determine possible differences between these two groups (Table 2). The presence of MFCs in patients with LN was not associated with any of the characteristics investigated (age at time of biopsy, time since SLE diagnosis, age at birth of male child, time since birth of first male child and number of male pregnancies). However, it is worth noting that in the group without MFCs, 40% of women were multiparous, almost twofold higher compared with primiparous women from the group with MFCs (22%) (Table 2). With regard to renal function, both groups presented with similar 24-hour proteinuria; however, women with MFCs had significantly lower levels of serum creatinine at the time of biopsy (with MFCs = 1.28 ± 0.73 mg/dl *versus* without MFCs = 3.35 ± 2.9 mg/dl; *P* = 0.03) (Table 2).

**Table 2:** **Pregnancy history, renal function and histological classification of renal biopsies of patients with lupus nephritis divided into groups containing renal biopsies either with or without male fetal cells** ^**a**^

**Characteristics**	**With MFCs**	**Without MFCs**	***P-value***
Number (%)	9 (64)	5 (36)	
Age at biopsy (yr), median (range)	38 (22 to 48)	41 (32 to 56)	NS
Time since SLE diagnosis (yr)	6.1 ± 7.6	9.4 ± 4	NS
Age at birth of the first male child (yr)	21.5 ± 3.9	22.6 ± 3.8	NS
Time since birth of the first male child (yr)	16.3 ± 7.7	21 ± 7.3	NS
Number of male pregnancies, n (%)			
1	7 (77.8)	3 (60)	NS
>1	2 (22.2)	2 (40)	NS
Serum creatinine (mg/dl)	1.28 ± 0.73	3.35 ± 2.9	0.03
Proteinuria (g/24 hr)	1.78 ± 1.2	1.15 ± 0.7	NS
Histological classification of renal biopsies^b^, n (%)			
Mesangial glomerulonephritis (class II)	1 (11.1)	0 (0)	NS
Focal proliferative glomerulonephritis (class III)	3 (33.3)	0 (0)	NS
Diffuse proliferative glomerulonephritis (class IV)	3 (33.3)	3 (60)	NS
Membranous glomerulonephritis (class V)	2 (22.2)	2 (40)	NS

^a^LN, Lupus nephritis; MFC, Male fetal cell; NS, Not significant. Data are given as mean ± standard deviation. ^b^World Health Organization classification scheme, as updated in 2003 by the International Society of Nephrology/Renal Pathology Society [16]. No class I or VI biopsies were found.

### Male fetal cells and the histological classification of lupus nephritis

We also investigated whether MFCs were associated with any specific histological features of LN. We found that the most common forms of glomerulonephritis among the study groups were diffuse proliferative glomerulonephritis (43%), followed by membranous glomerulonephritis (29%), focal proliferative glomerulonephritis (21%) and mesangial glomerulonephritis (7%). No biopsies were classified as class I or VI (Table 2). It must be noted that patients with LN without MFCs predominantly presented as glomerulonephritis class IV (60%) and class V (40%) (Table 2).

**Figure 1:**
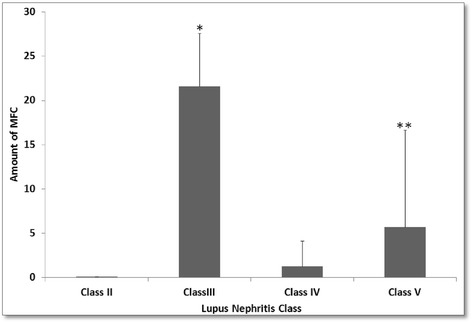
Amount of male fetal cells in renal biopsies and the associated histological lupus nephritis class. MFC, Male fetal cells. *P < 0.01 for class III *versus* class IV; **P < 0.05 for class III *versus* class V.

When associations between the amount of MFCs and histological LN class were investigated, we found a higher proportion of MFCs in LN class III than in classes IV and V (class III = 21.6 ± 6, class IV = 1.27 ± 2.9 and class V = 5.7 ± 11; *P* = 0.004) (Figure 1). Indices of activity and chronicity of LN were not correlated with the amount of MFCs.

## Discussion

Although fetal microchimerism is a common phenomenon in biology and is found in healthy women many years after pregnancy completion, only a few studies of its role in SLE have been conducted [21]. Most researchers have investigated the association of SLE and male microchimerism in whole blood, and most of the reports have confirmed the higher prevalence of MFCs in women with SLE than in controls [11,14,22,23]. However, studies showing the prevalence of MFCs in kidneys of patients with SLE and LN are much scarcer [12,13,24,25].

Our study shows a significantly higher prevalence (64%) of MFCs in kidney biopsies from women with SLE and LN than in the control group, in which no MFCs were found. No differences in pregnancy history were found between the LN and control groups. Neither could we find any correlation between pregnancy history and the amount of MFCs detected. However, there was a significantly higher amount of MFCs found in the group of patients with LN presenting with serum creatinine levels ≤1.5 mg/dl (Table 1).

Taken together, these data corroborate our previous study in which we detected increased MFCs in whole blood from patients with SLE and strongly suggest a role for these MFCs in the etiopathogenesis of LN. Furthermore, our results are in line with other studies in which authors reported an association between MFCs and LN [12-14,24,25]. In two different studies, Kremer-Hovinga *et al*. investigated the presence of the Y chromosome using fluorescence *in situ* hybridization (FISH). Microchimeric cells were found in 55% of renal biopsy samples from women with LN and in 25% of kidney autopsy specimens without histomorphologic lesions that served as controls [12]. In the second study, microchimerism was investigated in postmortem tissues from seven patients with SLE, and the researchers then compared these tissues with histologically normal organs from 34 control women. Chimeric MFCs were found in all seven patients and in 44% of the controls [24].

Khosrotehrani *et al*. could not find an association between microchimeric MFCs and SLE using the FISH method to detect the Y chromosome in skin tissue from women with SLE [26]. However, in a case report study of a woman with severe SLE, the same group of authors demonstrated the presence of a large number of MFCs in necropsy specimens from clinically affected tissues [25].

Although our results are generally in agreement with these previous studies, we did not find any microchimeric MFCs in our control group. This divergence may be the result of different techniques used to detect microchimerism and especially of the fact that strict exclusion criteria for pregnancy history and blood transfusion were not employed in the other studies.

Furthermore, comparisons of results between different studies should be interpreted with caution for several reasons. (1) Few reports are available, and only a small number of patients have been studied. (2) Different methods have been used to detect MFCs in different tissues. (3) There is a lack of strict inclusion and exclusion criteria to assess history of blood transfusion and pregnancy/abortion. (4) Control groups studied have included biopsies that varied from biopsies taken from patients with other kidney diseases to tissue specimens obtained postmortem. (5) The effect of SLE treatments on microchimeric cells is unknown.

Our results also suggest that LN can occur in the absence of microchimerism, because 36% of women in the SLE with LN group did not present MFCs in renal tissue (Table 2). Using different techniques, other researchers have reported similar prevalences (between 45% and 54%), and although MFCs can remain in the peripheral blood for years, it is unknown how long these cells could remain in the tissues [12,13,24].

Again, analysis of pregnancy history of patients with LN with versus without MFCs did not show significant differences between the two groups (Table 2). However, as observed earlier in the overall analysis of the women with LN, the group in which microchimerism was present had significantly lower levels of serum creatinine than the group without MFCs (Table 2).

The role of fetal microchimerism in SLE is controversial, raising the question whether these cells are targets or innocent bystanders [27]. One hypothesis is that microchimeric cells could provide a chronic source of foreign antigens, generating persistent inflammation and leading to injury and release of additional foreign antigens. As foreign antigens, these microchimeric cells could be involved in interactions between host and nonhost cells, similar to graft-versus-host disease after stem cell transplantation, and trigger autoimmune disease [28].

Another possibility is the repair hypothesis, wherein the microchimeric cells are involved in repair mechanisms and, in response to inflammation and chronic tissue damage, these cells are actively recruited to the injured tissue and help in the repair and regeneration process [28].

Also, the low levels of MFCs could be an advantage because they could be tolerated by the immune system and could be recruited to the damage tissue and aid in the repair process [27]. Additionally, microchimeric cells have been identified as being mesenchymal stem cells based on their morphology and immunophenotype. As a consequence, their capacity for transdifferentiation in tissue repair could alter the tissue microenvironment by secretion of soluble factors and amelioration of tissue damage in response to injury and disease [29,30].

Although renal biopsy plays a crucial role in the diagnosis of the specific form of LN, its role in predicting disease outcome has been controversial [18]. Therefore, we sought to find an association between the type of glomerular lesion in LN and MFCs. Contrary to Kremer-Hovinga *et al*., we found a similar distribution of WHO glomerulonephritis classes among patients with LN, although a trend toward a higher prevalence of class IV was observed (Table 2) [12]. Similar results were seen among patients in whom MFCs were present. In contrast, all five patients with LN without MFCs had only either class IV or class V glomerulonephritis. If we arbitrarily consider classes IV and V as the most severe stages of glomerular disease, these findings suggest a possible association between the severity of LN and the absence of MFCs, highlighting that the development of microchimerism could have a beneficial effect in LN [31].

Additionally, it was reported previously that elevated serum creatinine at the time of the kidney biopsy is associated with an increased risk of chronic renal failure [32].

As we found lower serum creatinine levels to be associated with a higher amount of MFCs and, contrarily, worse renal function in the group without MFCs, ours results are in line with the hypothesis that the presence of MFCs at the time of renal biopsy could have a protective role in LN. These findings also lend support to the possibility that these cells are tolerated by the immune system and that, in response to tissue injury, they would be recruited to the injured site and aid in the repair [27].

We do acknowledge that limitations of the present investigation are the small sample size studied and the inclusion and exclusion criteria used, which could introduce some bias when interpreting results. Probably, these limitations justify the scarce number of reports in this subject area in the existing literature. However, as far as we know, this is the first study in which a very sensitive molecular technique, real-time qPCR, was used to quantify MFCs in renal biopsy samples. Furthermore, to avoid false-positive chimerism due to undetected miscarriage and blood transfusions, we used a very strict questionnaire to interview all women included in the study, resulting in the detection of MFCs being consistently negative in our control group.

## Conclusions

Our data indicate a high prevalence of MFCs in renal biopsy specimens from women with LN, suggesting a role for these microchimeric cells in the etiology of LN. The present report also provides some evidence that MFCs could have a beneficial effect on the disease process. Nonetheless, the true biologic significance of MFCs and whether they act as protectors or insurgents in SLE and LN remain to be elucidated, and thus further multicenter studies are required.

## Abbreviations

FISH: Fluorescence *in situ* hybridization
LN: Lupus nephritis
MFC: Male fetal cell
qPCR: Quantitative polymerase chain reaction
SLE: Systemic lupus erythematosus
SRY: Sex-determining region Y
WHO: World Health Organization
